# Supporting Holistic Health and Gestational Diabetes Mellitus Risk Reduction Among Young Native Females Prior to Pregnancy: A Qualitative Exploration

**DOI:** 10.3390/ijerph22010025

**Published:** 2024-12-28

**Authors:** Sarah A. Stotz, Luciana E. Hebert, Lisa Scarton, Kelli Begay, Kelly Gonzales, Heather Garrow, Melanie Charley, Melanie Aspaas, Denise Charron-Prochownik, Spero M. Manson

**Affiliations:** 1 Department of Food Science and Human Nutrition, College of Family and Consumer Sciences, Colorado State University, Fort Collins, CO 80523, USA; 2 The Institute for Research and Education to Advance Community Health, Washington State University, Seattle, WA 99163, USA; luciana.hebert@wsu.edu; 3 Department of Family, Consumer and Health Sciences, College of Nursing, University of Florida, Gainesville, FL 32611, USA; lscarton@ufl.edu; 4 Independent Researcher, Oklahoma City, OK 73012, USA; kelli@mavencollectiveconsulting.com; 5 School of Public Health, Portland State University, Portland, OR 97201, USA; kgonzales@indigenous-equity.org; 6 Saint Regis Mohawk Diabetes Center for Excellence, Akwesasne, NY 13655, USA; heather.garrow@srmt-nsn.gov; 7 Independent Researcher, Portland, OR 97035, USA; melaniecharley71@yahoo.com; 8 Colorado School of Public Health, University of Colorado Anschutz Medical Campus, Aurora, CO 80045, USA; melanie.aspaas@cuanschutz.edu (M.A.); spero.manson@cuanschutz.edu (S.M.M.); 9 Department of Health Promotion and Development, School of Nursing, University of Pittsburgh, Pittsburgh, PA 15260, USA; dcpro@pitt.edu

**Keywords:** pre-pregnancy, Indigenous, American Indian and Alaska native, adolescent females, food and nutrition insecurity, holistic wellness, culturally centered, traditional foods, gestational diabetes risk reduction, diabetes risk reduction

## Abstract

AI/AN communities are disproportionately impacted by food insecurity and gestational diabetes mellitus (GDM). Decreasing the risk of GDM can interrupt the intergenerational cycle of diabetes in AI/AN families, and can decrease diabetes-related health disparities. The goal of this study was to explore ways of supporting holistic health and reducing the risk of GDM among young American Indian and Alaska Native (AI/AN) females prior to pregnancy. Semi-structured interviews were conducted with adult AI/AN women (>18 years) who had GDM or who have young female relatives (e.g., daughters) (n = 41), AI/AN females between 12 and 24 years (n = 18), and key experts in food/nutrition and health within AI/AN communities (n = 32). Three themes emerged: (1) guidance on how to support young females’ holistic wellness; (2) evidence that generations of colonial violence, including forced removal, forced poverty, and the imposition of a Western-based food system, causes deeper, systemic fracturing of traditional cultural food knowledge and practices; and the fact that (3) opportunities for improved adolescent female health are rooted in AI/AN values and how AI/AN people resist the impacts of anti-Indigenous racism through family-based, community-led, and holistic health. These themes suggest alternative understandings about the relationships between food insecurity and holistic pre-pregnancy health and can guide our next steps in decreasing health disparities in these communities.

## 1. Introduction

The traditional diets and methods of food acquisition (e.g., foodways) of American Indian and Alaska Native (AI/AN), Native Hawaiian, and other Indigenous peoples in the United States are inherently diabetes-preventative [1]. However, these traditional food practices, such as gathering, hunting, seed storage, and fishing, have been systematically destroyed because of colonization and ongoing settler colonialism. Colonization resulted in genocidal projects, and when that was unsuccessful, additional policies and practices were implemented to weaken Tribal Sovereignty and traditional, cultural practices. These include forced removal from traditional homelands, unfulfilled treaty responsibilities, superfund sites (e.g., locations polluted with hazardous materials) on or near reservation lands, and racist policies which negatively impact the health and prosperity of all people of color in the United States, including AI/AN, Native, and Indigenous peoples [2,3,4]. The destruction of traditional foodways have likely contributed substantially to the burden of gestational diabetes mellitus (GDM) among Native females, who are twice as likely to have GDM and a subsequent diagnosis of type 2 diabetes (T2D) than non-Hispanic white females [5]. GDM is a significant risk factor for both mothers and offspring developing T2D, creating an intergenerational cycle of diabetes in Native communities [6]. Reducing the risk of GDM in Native women is imperative to reducing diabetes health disparities among Native communities. A healthy diet can both prevent and help manage chronic diseases such as diabetes.

The United States Department of Agriculture (USDA) defines food insecurity as a lack of consistent access to enough food for an active, healthy life [7]. Food insecurity and limited access to healthful food can give individuals no choice but to rely on calorie-dense, carbohydrate-rich, processed foods, which negatively impact blood sugar in the general population [8,9] and Native populations alike [10]. As the adapted National Institutes of Minority Health Disparities Research Framework outlines [11], food insecurity is exacerbated in Native communities by water insecurity [12], stolen land and land-based resources, commodity foods [13], forced relocation, and environmental pollution, all of which have devastated their traditional healthy food practices [7,14]. Women deserve special consideration in discussions of food insecurity and its effects on health, nutrition, and behavior [15]. Specific to women of reproductive age, living in a food-insecure household may increase risk of greater weight gain and perinatal complications [16]. In adolescent females, food insecurity is associated with elevated BMI [17], increased depressive symptoms [18], and smoking [19], and is a strong predictor of poor pregnancy outcomes [18]. Reducing the risk of GDM for Native females prior to their first pregnancy may effectively decrease diabetes disparities among Native communities [6].

The current study sought to understand the perspectives of Native women, adolescent and young adult females, and key informants (e.g., healthcare providers, Native food/nutrition leaders) who serve these audiences, and to conduct a robust, strengths-based qualitative inquiry to answer the following research questions: How do Native girls and women, as well as experts in the field of food, nutrition, wellness, and Indigenous health, (1) perceive the relationship between food insecurity and holistic health for Native females? Further, it aimed to (2) describe wellbeing for Native females and explain what is needed to achieve this. Throughout this paper, we refer to “females” and “women” as inclusive of people who can become pregnant, regardless of their gender identity. We also refer to the priority audience as “Native” as inclusive of American Indian, Alaska Native, Native Hawaiian, and other Indigenous peoples, as preferred by the Native members of our research team.

## 2. Materials and Methods

### 2.1. Theoretical Framework

This qualitative study is theoretically supported by a constructivist approach [20], Traditional Ecological Knowledge and Ancestral Knowledge Systems [21], and an intersectional methodology [22]. The social constructivist perspective suggests that people are always developing meanings and understandings in social, cultural, and historical contexts. From this perspective, people construct, form, and negotiate subjective, complex understandings of food, eating, and health through their personal experiences and interactions with other people and their contextual environment. Concepts from both Traditional Ecological Knowledge and Ancestral Knowledge Systems support decolonizing research, redistributing power to traditionally marginalized communities, honoring and centering Indigenous ways of knowing and values, and challenging hegemonic Western research practices with critical consciousness [23]. Finally, intersectionality helps us to understand women’s (and others’) lived experiences as emerging from a range of intersecting identities and oppressions, and how these identities are shaped by interpersonal, institutional, structural, and representational forms of power [22,24]. Together, these frameworks informed our culturally relevant approach to sampling, data collection, and data analysis.

### 2.2. Study Participants, Recruitment, and Setting

**Figure 1:**
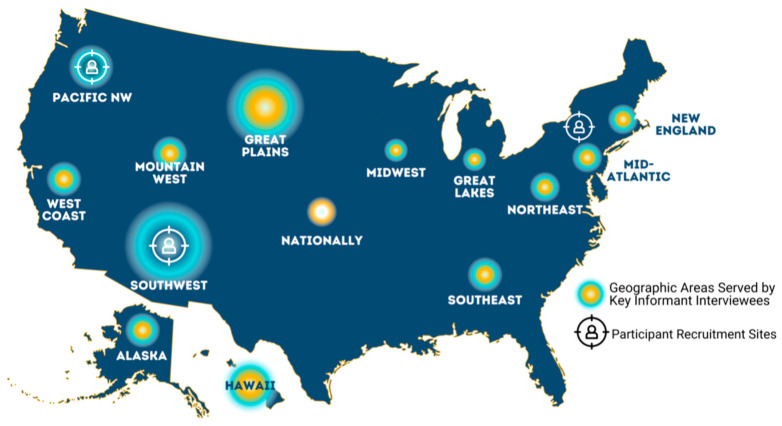
Map of the United States depicting geographic areas served by key informant interviewees and recruitment sites for Native focus group participants.

We used both maximum variation purposive and snowball sampling methods to recruit participants in different strata from across the country to participate in this qualitative study [25]. Locally situated, Native, female public health workers or healthcare providers in northwestern Oregon, upstate New York, and north central New Mexico recruited participants for focus group discussions. The participants were adult Native women who had a history of GDM or who have female relatives (e.g., daughters, nieces) between 12 and 24 years old (n = 41), and Native females between 12 and 24 years old (n = 18). Many of these adult and adolescent/young adult females participated in a randomized controlled trial, entitled “Stopping GDM in Daughters and Mothers”, in 2017–2019 [26]. Additionally, the research team recruited key informants with both Western and/or traditional expertise in food, nutrition, food systems, and reproductive, adolescent, and women’s health within Native communities (n = 32) from across the United States to participate in individual qualitative interviews. We used their own professional networks and literature reviews to seek researchers who have published in relevant topical areas and sought guidance from other Indigenous scholars to identify potential expert key informants. The research team sent email invitations to each potential key informant, and, at the end of each interview, asked interviewees to recommend additional individuals who might be interested or whose perspective would be important to include. We also recruited participants for healthcare provider focus groups through a listserv which includes past participants of an annual maternal/child health conference for providers who serve one Southwestern reservation. A map displaying the geographic representation of participants of the areas served by key informants can be found in Figure 1.

### 2.3. Data Collection and Procedures

As informed by our conceptual framework, and to decolonize this research and create an environment where Native women were comfortable and felt safe sharing their stories, we employed exclusively Native women to recruit and collect the qualitative data for the Native adult and adolescent female focus groups. Further, we used a semi-structured, trauma-informed conversation and strengths-based [27] moderator guide with probes which included scenario-based questions to avoid triggering or re-traumatizing the focus group participants. These scenario-based questions were specific to access to healthy food (e.g., food security) and were used as a means of decreasing feelings of shame related to experiencing food insecurity [28]. The moderator guide was developed by 5 qualitative researchers, 3 of whom are Native women themselves. Details of the moderator guide can be found in Table 1. We conducted separate focus groups of adult women with a history of GDM and/or who had daughters/nieces/ granddaughters in the priority age range (12–24 years old), and of Native adolescent and young adult females. The focus group methods intentionally offset the power dynamic between the researcher and the participants. Two PhD-trained female qualitative researchers, one a Native nurse and one a non-Native maternal health researcher, conducted the individual key informant interviews. Of note, though the “types” of participants were separated during these data collection procedures, as supported by the intersectionality framework [22], the researchers decided not to separate this dataset by “type” of participant, so as to intentionally triangulate these findings across key stakeholders. The interview guide is shown in Table 1.

All key informant interviews and focus groups with Native adult and adolescent females were conducted virtually (e.g., Zoom) for the convenience of the interviewees [29]. Of note, two other members of the research team, also qualitative researchers, conducted two in-person focus group discussions with healthcare providers at an annual maternal and child health conference for healthcare providers who care for reservation-based women and children in the southwestern United States. All interviews and focus groups were recorded; all data were collected between September 2022 and February 2023. Table 2 presents details of the data included in this study. To collect information about their background characteristics, each participant completed a brief descriptive demographic survey administered via Remote Electronic Data Capture (REDCap) at the end of their focus group or interview. All participants were offered USD 50.00 for their time in either a focus group or individual interview. We secured the required Institutional Review Board approval from both the Indian Health Board National IRB (#N22-N-04) and the Colorado Multiple IRB (#22-1018) prior to human-subject research commencing.

### 2.4. Analysis

De-identified audio files of interviews were transcribed verbatim by a professional transcription service. Prior to analysis, each transcript document was verified against the audio-file for accuracy. We employed a constructivist epistemological approach, using content analysis with thematic coding methods to guide analysis [30]. Using thematic coding methods, the data were coded in various quotation increments depending on the context of the quotation. The intersectionality methodology prompted us to consider the interrelationship between gender and Indigenous identity and acknowledge both the coloniality of gender and Indigenous people that impacts the participants’ lived experiences, known as “decolonial feminism”, throughout the analytic process [31]. Three trained qualitative researchers (one of whom is Native) coded the transcripts and consulted with two Native qualitative research experts throughout the analytic process. We used a multi-stage analytic process using both inductive and deductive coding. First, the lead author created a deductive codebook based on the moderator guide, Indigenous ways of knowing literature [21,23,32,33], and guidance from a Native qualitative expert and co-author on this manuscript. She then used this codebook and coded all of the transcripts using both deductive (e.g., from the codebook) and inductive coding (i.e., those that arose directly from the data). The researchers used a flexible codebook that allowed for inductive codes based on patterns from the data, rather than relying on deductive codes alone. After coding all of the transcripts once, the lead author then defined all of the codes and engaged the rest of the analytic team to double-code 20% of the transcripts. Using this coding strategy, the codes then evolved into a hierarchical code system, which led to the development of categories. An example of an inductive category (e.g., code group) was “Impacts of Anti-Indigenous Racism”, which was supported by the clustering of 23 codes including shame, grief, trauma, pollution, fatigue, etc. After all of the coding was complete, we used codes and categories to develop code networks. The code networks served as a visual network representation of the coded data, facilitated the identification of emergent themes, and eventually led to the most prominent themes. Additionally, we conducted member checking by recording a short presentation describing the key themes and sharing the presentation and slides with all of the participants (via email). The participants were invited to provide feedback during a 2-week period using an anonymous Google document or by contacting the lead researcher to discuss preliminary findings and provide feedback via phone or email or scheduled teleconference. Data management and analysis were conducted using Atlas.ti (Version 23.1.1) to digitize the analytic process. The analysts met on a bimonthly basis to discuss the analytic process. All three qualitative analysts wrote reflexive memos and revised them iteratively throughout the analysis [34].

## 3. Results

### 3.1. Participant Demographics

The background characteristics of the study participants stratified by participant type are shown in Table 3. Our final sample totaled 38 Native adult women who completed the survey and 21 Native adolescents who completed the survey. Among our final sample of 38 adult Native females, the mean age was 43 years (range 28–66 years). A plurality (n = 16; 42%) had at least a college education, and 24% reported they had diabetes. Adult Native females reported a mean household size of 4.5 persons, and over half (53%) reported that their household income was comfortable, while 45% reported they had just enough to make ends meet. Nearly all (n = 36; 97%) had been pregnant, and one-third (n = 12) reported being diagnosed with gestational diabetes. Over one-third (n = 12) reported being food-insecure; 24% (n = 9) reported receiving Supplemental Nutrition Assistance Program (e.g., SNAP, formerly known as food stamps) benefits; and 46% (n = 17) reported that their household receives reduced or free school lunch benefits. Adult Native females reported a mixture of sources for where their food typically came from, including the grocery store (100%), restaurants (83%) convenience stores (61%), hunting/fishing/gathering (50%), their own garden or yard (42%), a community garden (41%), school (38%), and other family members (36%).

Among our final sample of Native adolescent and young adult females (n = 21), the mean age was 20 years. None had diabetes, and while four (19%) had been pregnant, none reported ever being diagnosed with gestational diabetes. Over half (n = 11; 52%) reported that their household was food insecure, and the same number reported that their household had just enough to make ends meet. Roughly a quarter (19–24%) of Native adolescent and young adult females reported they received each type of food benefit (e.g., SNAP). Native adolescent and young adult females also reported a variety of sources for the food in their household; compared to their older Native women counterparts, Native adolescent and young adult females reported higher percentages of obtaining their food from other family members (62%) and school (48%), and lower percentages of obtaining their food from hunting/fishing/gathering (30%), their own garden or yard (29%), and a community garden (10%).

Our final sample of key informants totaled 58 participants, 80% of whom were female and who ranged in age from 30 years to 76 years. Thirty-nine percent of key informants identified as AI/AN, and two (4%) identified as Native Hawaiian or Pacific Islander. Over half of key informants identified as white. A majority (n = 47; 84%) had a graduate degree.

### 3.2. Qualitative Findings

Three cross-cutting, salient themes emerged from this dataset. These include (1) guidance on how to support young females’ holistic wellness (e.g., strong cultural identity, physical activity, and support from multigenerational family and community); (2) the fact that generations of colonial violence, including forced removal, forced poverty, and the imposition of a Western-based food system of high-fat and sugary foods, causes deeper, systemic fracturing of traditional cultural food knowledge and practices; and (3) that opportunities for improved adolescent female health are rooted in Indigenous values and how Native people resist the impacts of anti-Indigenous racism. Throughout this section, we detail each theme and provide exemplifying quotations that support each theme.

Table 4 includes categories (derived from code clusters) that support each theme.

### 3.3. Theme #1

First, the participants provided guidance on how to support young females’ holistic wellness. The participants responded to moderator guide questions such as “describe a healthy adolescent female in your community” and “what do adolescent females need to be healthy”, which led to this theme. One Native key informant explained the need for mentors, resources, and a sense of ownership:

*I think they need mentors. I think they need support from one another, that’s a tough age. So I hope that they don’t have to deal with lateral oppression like our generation definitely does when they’re coming up. So I think they need just basically support and also resources, educational resources, the ability to experientially do these things on their own, ability for them to create media around these pieces. I think all those things heighten their sense of ownership. We don’t give them to them, they already own it, but heightens their sense of ownership and connection to their wellness and their wellbeing.*

(Native key informant).

Another Native key informant, who was also the mother of adolescent females, shared her thoughts on multilevel, holistic approaches to supporting Native adolescent females:

*They would have a strong relationship with their parents, grandparents, guardians, their male cousins and brothers would protect them and make them feel as if they’re special and not a burden. What else? There would be educational pathways that were adaptable to that person’s unique needs and talents and gifts and abilities. And there would be equitable pay for her. (…) I mean, access to good healthcare, that’s access to good healthcare. (…) Not trying to replicate the broken system in this country, in this community. It’s just drives me in saying it’s like, oh, why do we need to adhere to a standard that’s not even the best in the world? I don’t. And then I just think about the built environment. That environment is safe, it’s accessible.*

(Native key informant and mother of adolescent females.)

One Native adolescent female shared her thoughts on the importance of movement for mental wellness, and de-emphasized the focus on food:

*I know for me, it has a lot to do with movement. Here, (…) spending time around elders and peers. (…) Because if you’re going to work hard, you got to take care of yourself just as hard. And I think so much is focused on diet where that’s really important. But I find if I start with movement, then my mental health gets better. And if I remember to be social and I remember to get outside and do things that make me laugh and make feel good, then I want to feed myself better too. But the more I focus on food itself, it hasn’t made the impact in my life. But as much as connection and not just activity, working out, but joyful, something you love to be active in doing. That has helped me a lot.*

(Native adolescent female.)

Many participants discussed the importance of cultural connectedness to support Native adolescent wellness, as exemplified by this Native key informant:

*And in our culture, the healthiest youth that we have in our community for Native are the girls that understand their culture. Culture’s always a strength and it’s always associated with wellness, and you could even fact check that. I don’t know where to cite the sources, but they’re out there. Letting them have that cultural identity and that pride, whether it’s the dancing or however they express it, the art, I think those are the healthiest youth.*

(Native key informant.)

The participants provided guidance on how to support young females’ holistic wellness and also discussed the challenges to supporting holistic wellness in the context of the past and present-day implications of colonization, as elucidated in Theme #2.

### 3.4. Theme #2

The second theme focused on key barriers to healthful eating and healthy adolescent females. These barriers are rooted in generations of colonial violence and anti-Indigenous racism. Examples include forced removal, forced poverty, and the imposition of a Western-based food system of high-fat and sugary foods, which caused deeper, systemic fracturing of traditional cultural food knowledge and practices. Participants shared insights regarding the history of Native foodways and lasting implications, as discussed by this Native key informant:

*(...) Before, the food was taken away, their lands were taken away from them, or made to be toxic, so they lost access to a lot of their traditions. And then it was like the government is like, here, here’s some commodities foods. So that become really ingrained in a survival of people trying to survive. And then there’s a fondness for foods that come out of that. Indian tacos, for example, or fried bread is an example. And fresh vegetables weren’t served. So people, they didn’t grow up with that. And then that gets passed on and gets passed on. And it’s hard to... So I think all Natives have gone through.. [trauma]. (…) the way you think about eating is based on your grandparents, your parents, and what they were serving you.*

(Native key informant.)

Another Native key informant shared the implications of boarding school-related trauma:

*And for a lot of our generations, due to boarding schools, you just didn’t grow up with that. And so I sense, and this is what I’ve seen with some of the younger people, is there’s this sense of almost of shame that they don’t know how to do, prepare traditional foods, or don’t know a lot about their ancestral food connection. And I feel like those connections, and I always share in our outreach programs is, there’s nothing to be ashamed of. I mean, this is common due to the historical trauma, that you don’t know how to prepare your foods or understand your cultural foods, and ways and language.*

(Native key informant.)

A Native mother discussed her experience with body shaming and the lack of consistent Native female role models in her life:

*And I remember being younger and having my dad’s friend cut my hair and he goes, “We have to cut it some type of way to hide her round cheeks.” And that stuck with me for so long, and I was only eight years old. And now that I’m older, I see that I get these cheeks from my grandma, I get these cheeks from my Auntie [NAME]. So I think what would help is, and of course there’s not one answer, but what would help is I have embodied positivity for our people, having representation in schools for our people. (…) So I don’t know, just support for our young women, it’s difficult.(…) [They say] “Oh, well, go to your family, go to your elders.” A lot of us poor, traumatized young people, we don’t have family, and that’s the reason why we have so many traumas. So I don’t know, just community support and seeing people who look like you in your life.*

(Native woman with a history of gestational diabetes and mother to an adolescent female.)

Participants discussed policies and systems that make it hard to be healthy:

*I don’t know. I think the main thing is that there are just so many structural and historical set barriers. The way that everything is set up right now is just a guaranteed unhealthy person. You know what I mean? How hard it is to get to the grocery store, how hard it is to get clean water, how hard it is to get fresh fruits and vegetables. The way the system is set up is to truly create unhealthy adults. And I feel like just talking fruit and vegetables (…) everything that the US government has done, just to really get them to this place of food insecurity.*

(Key informant.)

Finally, one Native adolescent female shared her experience about multigenerational diabetes in her family:

*Yeah, my whole family on my mom’s side has had diabetes. Every single, I don’t know every generation on my mom’s side. So for my mom, I feel like it was, she had more weight on her shoulders to stop that, even though obviously it’s not just completely her decision. But so we were in, I would get my finger pricked just to make sure and when they said, oh you might have whatever it was called, like pre dia, you might be pre-diabetic, I was like, oh shoot, we need to get our stuff together, so that we can cut off this cycle now, instead of waiting until it’s too late.*

(Native adolescent female.)

Participants focused on upstream causes that challenge healthful eating and other healthful behaviors.

### 3.5. Theme #3

Participants shared ideas and recommendations about opportunities for improved adolescent female health to mitigate the challenges discussed in Theme #2. These recommendations are rooted in Native values and how Native people resist the impacts of anti-Indigenous racism. These recommended solutions are largely family- and community-based, led by community, and focus on holistic health as a goal rather than weight or diabetes. The participants described resistance by way of food sovereignty, as discussed here:

*What I’ve learned is that the other thing that you can’t separate from nutritional wellness is food sovereignty. And that for many Native and other Indigenous people, food sovereignty is how they see their route back to nutritional wellness. In other words, not focusing on a particular diet per se. A dietitian might give somebody with diabetes, a diabetes diet or high blood pressure diet and say, “This is how you should be eating.” Rather than that, what many Indigenous people believe is that they need to reinvigorate Indigenous or traditional foods, some of which they may not even know how to prepare, some of which may have been gone from their native cuisine for many generations, but they want to bring it back. And they believe (…) if they consume more whole foods and traditional foods for their communities and cultures, that is a big part of the route back to health. And food sovereignty is really the key to all of that, which is the ability to decide what they want to consume, to have access to foods, to have them affordable, et cetera.*

(Native key informant.)

The importance of multigenerational family support was emphasized, as discussed here:

*I think extended family. Extended family networks are really important. I just see that families come together regularly, and around ceremony, family comes together to support. When they gather foods, they go out as an extended family, so it’s relationships. It’s knowledge exchange.*

(Native mother.)

One Native key informant who is also the grandmother to adolescent females shared her thoughts on the importance of food connecting Native people to their culture, and that this connection supports health and wellness:

*Even though I am a dietician by training, so I was trained in foods, have proteins and fats and carbohydrates, but what we understand more from an Indigenous point of view is that foods connect us to our history. They connect us to our spirit, they connect us to our grandparents, they connect us to our culture, they connect us to our environment, they connect us to our stories. And these are the connections that, when broken, make us unhealthy. And so we strive in our program to use foods that really help to solidify these connections, so that when you’re passing down your stories of who you are, when you’re sharing food with a neighbor, when you’re eating foods that your grandparents passed onto you, these are how you become healthier. That’s the overall basis of the work I do is using foods to strengthen the connection that make you healthy.*

(Native key informant and grandmother to adolescent females.)

Participants emphasized the importance of female mentors, role models, and multigenerational, circular thinking in promoting holistic wellness, as discussed here:

*No, I mean I just think we still have more work to do. I mean think it’s, again, it’s the whole system and very much a cyclical thing. It’s not a linear way of addressing it. And if we want, I guess the thing to think, and this is... Okay, this is what I think is so cool about being a woman. And this is the thing, the one thing that we got to make sure to pass on to our daughters. And I didn’t really appreciate this until one of my kumus, my mentors, told me this, that the generations, we carry the generations in our womb. If you think about that, if you think how much kuleana and responsibility we have and just knowing that all of our generations are here within us. If we can get that message out in what we do and just this whole idea of holistic and wellbeing, we want to take care of ourselves now because we know it’s going to take care of the future us. And it’s all within women, not men, within women. But we don’t talk about that in classes and stuff like that. And I think if we kind of made that connection, because it’s not a very western way of thinking, you know what I mean? But when we think about we have three pikos, and one of our pikos is that, is the fact that our future generation comes from there. And I know lots of native communities have that same thinking. So we just need to do a better job of weaving those things in together and just more female empowerment.*

(Native key informant.)

These recommendations provide community-based insight into ways to support young Native female’s holistic health.

## 4. Discussion

The original intent of this study was to explore the relationship between food insecurity and GDM risk reduction for Indigenous adolescent and young adult females. However, participant responses to interview questions and subsequent themes were far more focused on holistic health (vs. diabetes prevention) and further historical–contextual and upstream causes of challenges to holistic health (vs. present day food insecurity). Guided by an intersectional methodology, if a research question did not ask the “right” question relevant to the participants, we did not disregard our original research question, but rather included the participant-driven issues/themes as of equal importance [22].

Connection to traditional culture as a key facilitator in mitigating the effects of anti-Indigenous racism and the lasting impacts of colonization is well documented by Indigenous scholars [35,36,37]. These findings suggest that adolescent females who exhibit holistic health and opportunities to improve adolescent female health are rooted in connection to culture and relationality among family, community, and the environment (e.g., land) [27,33]. As supported by the Traditional Ecological Knowledge and Ancestral Knowledge Systems frameworks/theories, Indigenous values were also clearly identified across interviews as tenets which support adolescent holistic health and wellbeing [21,23,36]. Our results are consistent with prior studies with Native populations who have endured near extinction of their ancestral ways of life, culture, and identity as they were forcibly removed from their ancestral lands by US government policies [38]. For example, Lewis and colleagues [39] reported their work with American Indians in Oklahoma and the primacy of a common cultural context to effectively promote health benefits throughout an intensive cultural-based activity known as the “Remember the Removal” program [39]. The researchers described how physical, mental, and dietary changes and enhanced cultural learning, resilience, and traditional values were significantly improved after completion of the Remember the Removal program [39]. Other diabetes prevention programs, such as Cherokee Choices [40], Little Earth Strong [41], Tribal Turning Point [42], Stopping Gestational Diabetes in Daughters and Mothers [26], and hypertension prevention programs such as a hula-based program in Hawai’I [43]—among many others—focus on culturally centered approaches to disease prevention and health promotion. Despite the promising results of these studies, culturally centered health interventions remain relatively rare. A recent scoping review examining multilevel diabetes prevention and treatment interventions for Native people in the USA and Canada identified 10 interventions, 8 of which focused on youth, and the majority of which focused on just one level of intervention [44].

Barriers to holistic adolescent health include addressing trauma and the implications of intergenerational trauma as suggested by Walters and Simoni in their Indigenist stress coping model [32]. In their modified stress-coping model, the authors posit that the effect of chronic life stressors, such as historical trauma, on health is attenuated by cultural factors that function as buffers [32], as was clearly expressed by participants in our study. Specific to women’s health, both daughters and mothers told their stories of body image, mental health, the need to prioritize self-care, and how ‘balance’ was essential to achieving wellness. Considering intersectionality and the multiple identities (e.g., female and Indigenous) of most of these participants, the concepts of “decolonial feminism” [31] are particularly aligned. In Audre Lorde’s 1988 book, *A Burst of Light*, she wrote “caring for myself is not self-indulgent, it is self-preservation, and that is an act of political warfare” [45]. These words are salient across the discussions of how to support Native adolescent females. Participants in this study did not focus on weight, obesity, or weight loss as markers of adolescent female health, but rather were far more focused on self-care specific to mental health, healthy relationships, support networks, physical activity to connect with nature, and cultural identity. All of these discussions are inherently rooted in Native values and ways of knowing [21,23,36].

The absence of emphasis on body weight across the interviews is particularly informative for healthcare providers, public health officials, and researchers who hope to create resources and interventions to improve Native female adolescent health. Additionally, adolescent females are highly susceptible to body dysmorphia and disordered eating, especially those who live in food insecure environments [46], and weight-focused programming may cause more harm than healing. Historical trauma and anti-Indigenous racism [47] manifests in barriers to healthful eating in many ways—one challenge being that colonized food (e.g., commodity foods) is intimately linked to intergenerational survival and coping, and tastes/food preferences have been acculturated to these colonized (e.g., high-fat, high-sodium, high-sugar, processed foods) diets. Solutions to disrupting such intergenerational habits and preferences, while honoring the resilience of Native communities, include the following: investment in place-based, community-driven, Indigenous food sovereignty efforts and economies, and relationship building with traditional food teachings (e.g., gardening, gathering, farming, community gardens, seed and land stewardship, hunting, fishing, and food preparation). Though food insecurity is linked to adverse health for adolescent females (e.g., disordered eating) [46] poor maternal outcomes (e.g., GDM) [16], and other chronic diseases [48], the participants in this study did not focus on food insecurity, in and of itself, as a unique challenge to their wellbeing. Further, though a large proportion of mothers and adolescent daughters screened positive for food insecurity (34.2% and 52.4%, respectively), conversations on key barriers to health focused far more upstream than food insecurity (e.g., the causes of the cause). It may be that food insecurity was mitigated in some of the participants’ households by participation in federal food aid programs such as SNAP, Food Distribution Program on Indian Reservations (FDPIR), or the Special Supplemental Nutrition Program for Women, Infants, and Children (WIC). Additionally, though GDM has intergenerational implications for T2D disparities in Native communities, participants in this study did not focus discussions on diabetes prevention, and so it may be prudent for future interventions to focus on promoting holistic wellness and not explicitly aim to ‘prevent diabetes’.

Differences across the various groups (e.g., experts, adult women, adolescent females) are likely largely attributed to their positionality and lived experience. The intersectional framework suggests six core ideas including inequality, relationality, power, social context, and social justice, all of which are interconnected and allow researchers to compare and deconstruct oppression to understand intersectional inequalities [22]. In this study, the various groups (e.g., key informants, Native adult women) overlapped as data collection unfolded, and throughout the analytic process, it became clear that many of the “key informants” were also Native women who had adolescent female daughters, granddaughters, nieces, etc., and therefore their positionality reflected that of both “types” of participants in this project. Other differences in the way participants responded to the moderator guide questions were explained by their contextual positionality and how they were included as participants in this study. For example, the two healthcare provider focus groups were held as an optional breakout session at a maternal and child health conference for healthcare providers who serve Native women and children in the Southwest United States. Because of the nature of this conference, and the reason these individuals were gathered, much of these conversations focused on access to healthcare and reproductive health resources. Given most of these healthcare providers were not Native themselves, and the Native mothers, adolescent females, and Native key informants did not discuss healthcare access at any notable length, we did not emphasize healthcare access as a key point of the overarching themes. These participants responded to moderator guide questions largely with a Western healthcare lens. This is contrasted with many of the key expert interviewees who focused conversation on community-based, grassroots public health nutrition concerns. Despite these different orientations and contexts, the common threads across these differences are strongly represented in the three overarching themes. Another key difference included the vantage point by which participants discussed examples of healthy adolescents and how to help adolescent females achieve health. For example, across participant groups, there were discussions about the importance of healthy family mealtimes. Mothers focused more on discussing challenges related to making healthy meals (e.g., being busy, family members ‘on the go’ to sports/activities, and being exhausted from working all day), whereas key experts who were not mothers themselves focused more on challenges related to the colonized food system. Nevertheless, the overarching themes were salient across groups and, therefore, as supported by the intersectionality framework [22], the researchers decided not to separate this dataset by “type” of participant so as to intentionally triangulate these findings across key stakeholders.

### Limitations

This study has limitations which should be noted. First and foremost, by design, it is a qualitative inquiry and, therefore, cannot point to causal mechanisms, and is limited in terms of generalizability. Second, our original research questions were appropriate given the parent study and the funding for this project; however, as we spoke to participants, it became clear that food insecurity and GDM risk were merely one of a larger constellation of issues that affect Native adolescent females’ health and wellness. Finally, we did not collect data on rural vs. urban dwelling or tribal affiliation, nor did we separate options for participants to choose American Indian or Alaska Native (e.g., they were one category), so it may be difficult for readers to see their community reflected in these findings. Despite these weaknesses, our sample was broad and included groups of participants and key informants from across the US, including Alaska and Hawaii, which increases confidence in the commonality of our three overarching themes. Furthermore, we embraced this study’s theoretical orientations and adapted our research questions as guided by participant responses. Our inclusion criteria for “experts” included both Western and traditional trained experts in the key topic areas (e.g., women’s health, nutrition, and food access). Finally, our intentional research team of predominantly Native women offered a safe and collegial space for the Native female participants in this study.

## 5. Conclusions

The themes from this study suggest alternative understandings about the relationships between food insecurity and GDM risk within the social context of colonialism. The findings suggest the promise of relationship building to strengthen cultural identity and promote wellness opportunities that extend beyond the individual and influence social determinants that drive health. Given the intergenerational implications of GDM in Native populations, it is prudent that public health and healthcare organizations work with Native communities to support healthful environments and culturally relevant resources conducive to achieving holistic health among Native adolescent and young adult females to decrease the incidence of GDM and subsequent T2D to decrease diabetes health disparities.

## Figures and Tables

**Table 1:** Moderator guides used for qualitative data collection.

Type of Participants	Moderator Guide Questions
Native woman with history of GDM, Native mother of adolescent female, or Native adolescent/young adult female	Tell me about a food or meal that makes you feel good. *(Probes: content, location, company, emotions)* When you think of wellbeing, what does that look like for you? *(Probes: holistic, mind, body, emotional, spiritual, resources needed, location, facilitators)* Tell me about food in your community or family. *(Probes: meals, grocery shopping, location of meals, restaurants.)* What helps adolescent (teenage) females eat foods that support wellbeing. *(Probes: traditions, environment, finances, education.)* If your aunty was giving you guidance, what would she say about eating well so you can be your best self for your culture, community and future generations? What would she tell you if money was tight? What would you recommend for a Native mother in your community who was struggling to eat healthy because money is tight? *(Probes: resources, education.)* If a new Native family moved to your community and was having trouble getting enough food every week, what are the resources and options they would have? *(Probes: organization, programs, location of programs.)* Now that we’ve talked about food in your community, tell me about COVID-19 and food in your community.
Key informants (e.g., healthcare providers, traditional healers, community-based leaders, food/nutrition experts, food sovereignty and food security experts, reproductive health specialists, etc.)	Tell me about food and nutrition in your community. *Probe: what do folks in your community eat?* What does ‘holistic health’ or ‘wellness’ look like for adolescent females in your community? Describe a family in your community, which includes at least one adolescent girl, who exhibits health/wellness. *(Probes: facilitators, supports.)* What do teen and young adult girls need to support healthy eating? *(Probes: facilitators, strengths, resources.)* What types of resources or programs would help foster healthy eating for adolescents who live in food-insecure homes? *(Probes: financial support, federal program, local resource.)* Tell me about your current position/role as it relates to the topics we discussed and how you learned what you need to know to hold this position/role. *(Probes: education, training, title at place of work.)* Please let me know if there is anything else you’d like to share with me on these topics.

**Table 2:** Details of qualitative data collected.

Type of Participant	Number of Participants	Number of Focus Groups	Size of Focus Group (Mean, Range)	Length of Interviews/Focus Groups (Mean, Range in Minutes)
Individual key informant interviews	41	N/A	N/A	42.8 (26–73)
Key informant focus group	17	2	8.5 (7–10)	59.5 (51–68)
Adult Native female focus groups	38	9	4.4 (1–7)	63.0 (31–79)
Adolescent and young adult Native female focus groups	21	5	3.6 (2–5)	50.8 (37–67)

**Table 3:** Socio-demographic, household-, health history-, and food security-related characteristics of study participants, by participant type.

	Native Females (n = 38)	Native Adolescent and Young Adult Females (n = 21)	Key Informants (n = 58)
	n (%)	n (%)	n (%)
Sex *			
Male	0 (0)	1 (4.8)	11 (19.6)
Female	38 (100.0)	19 (90.5)	45 (80.4)
Prefer not to answer	0 (0)	1 (4.8)	0 (0)
**Age (years)**			
Mean, SD	43.3 (8.7)	19.6 (3.7)	51.8 (11.9)
Range	(28–66)	(12–25)	(30–76)
**Race ^+^**			
American Indian or Alaska Native	38 (100.0)	21 (100.0)	22 (39.3)
Asian	0 (0)	0 (0)	3 (5.4)
Black or African American	0 (0)	2 (9.5)	0 (0.0)
Native Hawaiian or Pacific Islander	0 (0)	0 (0)	2 (3.6)
White	2 (5.3)	0 (0)	32 (57.1)
Other	0 (0)	0 (0)	2 (3.6)
**Hispanic ethnicity**	1 (2.9)	2 (8.3)	3 (5.5)
**Education**			
High school/General Educational Development (GED) or less	10 (26.3)	16 (76.2)	2 (3.6)
Associate’s/Technical degree	12 (31.6)	0 (0)	1 (1.8)
Bachelor’s degree	13 (34.2)	5 (23.8)	6 (10.7)
Graduate degree	3 (7.9)	0 (0)	47 (83.9)
**Household size, mean (SD)**	4.5 (1.9)	4.2 (1.5)	
**Have diabetes?**			
Yes	9 (23.7)	0 (0)	-
No	26 (68.4)	16 (76.2)	-
Do not know	3 (7.9)	5 (23.8)	-
**Household income status**			
Comfortable	20 (52.6)	6 (28.6)	-
Just enough to make ends meet	17 (44.7)	11 (52.4)	-
Do not have enough to make ends meet	1 (2.6)	1 (4.8)	-
Do not know	0 (0)	3 (14.3)	-
**Ever been pregnant**	36 (97.3)	4 (19.1)	-
**Ever had gestational diabetes**	12 (33.3)	0 (0)	
**Household food security**			
Food secure	25 (65.8)	10 (47.6)	-
Food insecure	13 (34.2)	11 (52.4)	-
**Anyone in household receives**			
SNAP/food stamps/EBT	9 (23.7)	4 (19.1)	-
Food Distribution Program on Indian Reservations (FDPIR)/Commodity foods	6 (16.2)	5 (23.8)	-
Special Supplemental Nutrition Program for Women, Infants, and Children (WIC)	6 (15.8)	5 (23.8)	-
Free and reduced school lunch	17 (46.0)	4 (19.1)	-
**Where your food typically comes from**			
Grocery/supermarket	38 (100.0)	21 (100.0)	-
Own garden/yard	15 (41.7)	6 (28.6)	-
Convenience store	23 (60.5)	13 (61.9)	-
Community garden	15 (41.7)	2 (10.0)	-
Other family members	13 (36.1)	13 (61.9)	-
Neighbors/friends	7 (20.0)	5 (23.8)	-
Foodbank/pantry	7 (19.4)	6 (30.0)	-
School	14 (38.9)	10 (47.6)	-
Place of employment	6 (17.1)	4 (20.0)	-
Restaurant/eating out	30 (83.3)	17 (85.0)	-
Hunting/fishing/gathering	18 (50.0)	7 (35.0)	-
Other	3 (8.8)	1 (5.0)	-

* While female sex was a criterion for participation, one participant responded that they identified as male on the self-administered survey; ^+^ participants could select more than one race; SD = standard deviation.

**Table 4:** Categories supporting each overarching theme.

Theme	Supporting Categories
#1—Participants provided guidance on how to support young females’ holistic wellness.	Diet and nutrition education; Empower and advocate; Improve access to healthcare; Improve school lunch; Mental health support; Physical activity; Role models (Indigenous women, coaches); Safety and protection; Strong cultural identity; Support from multigenerational family and community; Support healthy communities and families.
#2—Key barriers to healthful eating and healthy adolescent females are rooted in generations of colonial violence and anti-Indigenous racism. Examples include forced removal, forced poverty, and the imposition of a Western-based food system of high-fat and sugary foods causes deeper, systemic fracturing of traditional cultural food knowledge and practice.	Colonized food system; Diabetes; Fatigue, exhaustion; Grief, loss; Intergenerational unhealthy eating/coping/acculturation; Pollution; Poverty; Racism; Shame; Stress; Trauma.
#3—Opportunities for improved adolescent female health are rooted in Native values and how Native people resist the impacts of anti-Indigenous racism. These solutions are family- and community-based, led by community, and focus on holistic health as a goal rather than weight or diabetes.	Food sovereignty; Healing; Relationships; Resilience; Self determination; Community-led solutions; Spiritual practices; Values (Indigenous): ○ *(listed are supporting codes)* ▪ Balance; ▪ Ceremony; ▪ Collective vs. self; ▪ Community; ▪ Connection to culture/cultural identity; ▪ Extended family; ▪ Family and social meals; ▪ Holistic health; ▪ Land; ▪ Language; ▪ Matriarchy; ▪ Nature; ▪ Relationality; ▪ Sharing; ▪ Storytelling; ▪ Teaching from elders; ▪ Traditional foodways.

## Data Availability

The data described in this manuscript will not be made available as they were collected from tribal community members, and it is the decision of the tribe and/or tribal-serving oversight entities as to how data are shared (or not) given the sovereign recognition of tribes and their data ownership.
